# How to Improve Prognostication in Acute Myeloid Leukemia with *CBFB-MYH11* Fusion Transcript: Focus on the Role of Molecular Measurable Residual Disease (MRD) Monitoring

**DOI:** 10.3390/biomedicines9080953

**Published:** 2021-08-03

**Authors:** Annalisa Talami, Francesca Bettelli, Valeria Pioli, Davide Giusti, Andrea Gilioli, Corrado Colasante, Laura Galassi, Rachele Giubbolini, Hillary Catellani, Francesca Donatelli, Rossana Maffei, Silvia Martinelli, Patrizia Barozzi, Leonardo Potenza, Roberto Marasca, Tommaso Trenti, Enrico Tagliafico, Patrizia Comoli, Mario Luppi, Fabio Forghieri

**Affiliations:** 1 Section of Hematology, Department of Medical and Surgical Sciences, University of Modena and Reggio Emilia, Azienda Ospedaliero-Universitaria di Modena, 41124 Modena, Italy; annalisa.talami@gmail.com (A.T.); francesca.bettelli@unimore.it (F.B.); pioli.valeria@aou.mo.it (V.P.); davide.giusti@unimore.it (D.G.); gilioli.andrea@aou.mo.it (A.G.); colasantecorrado@gmail.com (C.C.); l.galassi1591@gmail.com (L.G.); r.giubbolini10@gmail.com (R.G.); hillary.catellani@gmail.com (H.C.); donatelli.fran@gmail.com (F.D.); rossana.maffei@unimore.it (R.M.); silvia.martinelli@unimore.it (S.M.); patrizia.barozzi@unimore.it (P.B.); leonardo.potenza@unimore.it (L.P.); roberto.marasca@unimore.it (R.M.); 2 Department of Laboratory Medicine and Pathology, Unità Sanitaria Locale, 41126 Modena, Italy; t.trenti@ausl.mo.it; 3 Center for Genome Research, Department of Medical and Surgical Sciences, University of Modena and Reggio Emilia, Azienda Ospedaliero-Universitaria di Modena, 41124 Modena, Italy; enrico.tagliafico@unimore.it; 4 Pediatric Hematology/Oncology Unit and Cell Factory, Istituto di Ricovero e Cura a Carattere Scientifico (IRCCS) Policlinico San Matteo, 27100 Pavia, Italy; pcomoli@smatteo.pv.it

**Keywords:** acute myeloid leukemia, *CBFB-MYH11* fusion transcript, molecular measurable residual disease monitoring, prognostic thresholds and timepoints, intensive chemotherapy, clinical outcomes

## Abstract

Acute myeloid leukemia (AML) carrying inv(16)/t(16;16), resulting in fusion transcript *CBFB-MYH11*, belongs to the favorable-risk category. However, even if most patients obtain morphological complete remission after induction, approximately 30% of cases eventually relapse. While well-established clinical features and concomitant cytogenetic/molecular lesions have been recognized to be relevant to predict prognosis at disease onset, the independent prognostic impact of measurable residual disease (MRD) monitoring by quantitative real-time reverse transcriptase polymerase chain reaction (qRT-PCR), mainly in predicting relapse, actually supersedes other prognostic factors. Although the ELN Working Party recently indicated that patients affected with *CBFB-MYH11* AML should have MRD assessment at informative clinical timepoints, at least after two cycles of intensive chemotherapy and after the end of treatment, several controversies could be raised, especially on the frequency of subsequent serial monitoring, the most significant MRD thresholds (most commonly 0.1%) and on the best source to be analyzed, namely, bone marrow or peripheral blood samples. Moreover, persisting low-level MRD positivity at the end of treatment is relatively common and not predictive of relapse, provided that transcript levels remain stably below specific thresholds. Rising MRD levels suggestive of molecular relapse/progression should thus be confirmed in subsequent samples. Further prospective studies would be required to optimize post-remission monitoring and to define effective MRD-based therapeutic strategies.

## 1. Introduction

The latest 2017 European Leukemia Net (ELN) recommendations for the diagnosis and management of acute myeloid leukemia (AML) in adults [1] confirm AML with inv(16)(p13.1q22) or t(16;16)(p13.1;q22) as a single entity in the category of AML with recurrent genetic abnormalities. Together with AML with t(8;21)(q22;q22.1), they are collectively referred to as core-binding factor (CBF) AML, cytogenetically and molecularly defined by abnormalities involving genes encoding subunits of core-binding factors. CBFs are a family of heterodimeric transcriptional elements implicated in the regulation of hematopoiesis [2,3], containing a common CBFB subunit associated, in lymphoid and myeloid tissues, with RUNX1, one of the three CBFA members. The translocation (8;21) results in the creation of a chimeric gene *RUNX1/RUNX1T1*, while inv(16), or with significantly lower incidence t(16;16), leads to the fusion of the CBFB gene with MYH11, the smooth muscle myosin heavy chain gene, resulting in the chimeric *CBFB-MYH11* gene, which occurs in approximately 8% of adults with de novo AML [4].

According to 2017 ELN risk stratification [1], CBF AML are classified in the favorable risk category, with high CR rates after standard induction therapy and encouraging outcome, in particular after consolidation regimens containing a repetitive cycle of high-dose cytarabine [5]. Nevertheless, the 5-year overall survival (OS) rate in patients with CBF AML is about 50–60% [6], suggesting that it would be required to detect markers of more aggressive disease phenotypes, in order to optimize prognostic stratification-oriented treatments.

The effects on the long-term outcome of secondary cytogenetic abnormalities, detected in approximately 40% of inv(16) AML patients [7,8], and additional molecular lesions, which have been demonstrated to be required for leukemogenic transformations [9], remain controversial. 

In the last decades, probably depending on the accessibility of increasingly sensitive biomolecular tools, the focus has shifted to disease evaluation in terms of the dynamic quantitative assessment of molecular measurable residual disease (MRD). Indeed, the changing of MRD levels throughout cycles of therapy, in particular the reduction at a specific timepoint compared to pre-treatment baseline levels, has proved to be the most useful independent prognostic variable for survival, allowing one to identify patients at high risk of relapse, as possible candidates for more intensive therapeutic approaches, including allogeneic hematopoietic stem cells transplantation (allo-SCT).

The purpose of this manuscript is to offer an overview on the most prognostic factors affecting the clinical outcomes of patients with AML harboring *CBFB-MYH11*, with a special focus on the role of MRD monitoring in risk stratification and treatment guidance. 

## 2. Patient- and Disease-Related Features at Diagnosis

In general, AML with inv(16)(p13.1q22) or t(16;16)(p13.1;q22), hereafter referred as inv(16), exhibits some peculiarities, which also differentiate it when compared to AML with t(8;21)(q22;q22.1), abbreviated t(8;21) [6,7,8,10], such as presentation with acute myelomonocytic leukemia bone marrow morphology accompanied with abnormal/dysplastic eosinophils. Extramedullary disease, such as lymphadenopathy, hepatosplenomegaly, skin and gingival infiltrates, is often associated with inv(16) [6,8], whereas myeloid sarcoma in other extramedullary sites appears more frequently in t(8;21), and associated with worse prognosis [11]. 

Focusing on clinical variables (Table 1) [6,7,8,10,12,13,14,15,16,17,18,19,20,21,22,23,24,25,26,27,28,29,30,31,32,33], age is recognized as a negative prognostic factor, not only for a lower response to induction therapy, but also with regard to survival. CBF AML is relatively more incident among younger patients, accounting for only 5–8% of all AML over 60 years [34,35]. However, among the entire population, the incidence of CBF AML increases with age, reflecting the rise of all AML cases in the general population [22]. While elderly patients with CBF AML retain better prognosis compared to those with other AML subtypes, in comparison to younger patients with CBF AML, they reveal significantly worse outcomes. First of all, comorbidities and poorer performance status might hamper management with a standard regimen of therapy. As a consequence, older patients show high therapy-related mortality [8] because of excessive toxicity from chemotherapy. Nonetheless, it is demonstrated that when patients who die early within the end of induction are excluded, trends of long-term survival are similar in younger and older subgroups [22]. In addition, elderly patients seem to fail to clear leukemic cells, suggesting a refractory attitude, due to modifications in pharmacokinetic and multidrug resistance phenotype [36,37,38]. It has also been proposed that in older patients AML results from a series of mutational events, leading to the development of multiple subclones potentially showing chemo-escape mechanisms. Of interest, when treated with intensive schedules, elderly CBF AML patients frequently obtain CR, also in the setting of rescue therapy after relapse [23]. However, regarding long-term survival, the reported negative impact of age on OS and RFS is attributable to more frequent additional chromosome abnormalities, a possible indicator of genetic instability, as well as to attenuated post-induction treatments [39]. As evidenced in Table 1, some studies identified statistically significant age cut-offs; when considering data collectively, patients over 40 years of age have a dismal outcome.

As reported in several studies, WBC counts and parallel bone marrow/peripheral blood (BM/PB) blast percentages are higher in inv(16) than t(8;21) AML, at diagnosis [6,7,8,40], proposing a somewhat different type of proliferation kinetics, perhaps related to additional genetic aberrations, such as receptor tyrosine kinase (RTK*)* mutations [19]. Several authors agree on granting a prognostic relevance to white blood cell (WBC) counts, by recognizing, in some cases, cut-off points for statistical significance [10,13,17,29]. Nevertheless, the negative impact of leukocytosis in terms of either response to therapy or of long-term remission has not so far definitely assessed. Indeed, while a correlation between induction failure and early death in inv(16) AML emerging with hyperleukocytosis [8,10] has been documented, on the other hand, prolonged CR could also be observed regardless of WBC counts at onset. Furthermore, even if an unfavorable effect of high WBCs in increasing relapse rate has been demonstrated [41], no influence on OS was observed, probably depending on deep responsiveness to alternative rescue strategies. Analogous observations could be provided about platelets (PLT) count: the French AML intergroup [10] identified an optimal PLT count threshold predictive of induction failure, without worsening the subsequent risk of relapse. Although a documented WBC threshold indicating high risk is not currently available [42], data recommend more prudent approaches in induction treatment for patients admitted with leukocytosis and/or thrombocytopenia.

Hoyos et al. [17] confirmed both age and WBC count as variables associated with decreased OS, when using these parameters to separate three groups with statistically significant difference of survival, namely, 80% at 5 years for patients without adverse factors versus 61% for patients with one factor and 36% for patients older than 50 years and with WBC > 20 × 10^9^/L. 

A marginal role is conferred to sex and ethnicity. In fact, only Marcucci et al. [6] and Paschka et al. [43] attributed a prognostic significance to sex. The former study showed that male patients could survive longer, and the latter study reported that female patients with inv(16), younger than 60 years, were more likely to maintain shorter remission. Fewer reports addressed the prognostic impact of ethnicity. Inv(16) AML is somewhat less frequent among non-whites compared to patients with t(8;21). Moreover, Black and Hispanic patients have worse survival rates compared to white Caucasian patients [22]. These findings are possibly related to different biological behavior of the disease, because the environmental disparities are not considered sufficient to justify an evident divergence among patients treated according to the same protocols.

### 2.1. Secondary Additional Genetic Abnormalities

According to the latest evidence [30] formulated on the theory of multistep outset of AML [3,44], CBF AML seems to result from the acquisition of a sequential order of mutations, affecting firstly transcription and differentiation (such as CBF) genes, followed by activating alterations that increase proliferation, such as class III RTKs. Additional lesions in this latter gene family are the most common in CBF AML [30], involving primarily the *KIT* gene (occurring in 17–38% of CBF AML [45]), whose significance on the prognosis of AML has been widely debated. *KIT* aberrations could affect either the tyrosine kinase domain, with substitution of a single amino acid at codon 816 or 822 within exon 17 (higher incidence among CBF AML [14,28]), or the extracellular domain, corresponding to insertions or deletions in exon 8; more rarely, internal tandem duplications in exon 11 could impair the juxtamembrane domain. Due to the debatable negative impact attributed to *KIT* mutation, CBF AML with *KIT* mutation was formerly assigned to the intermediate category, whereas more recent guidelines only stated that prognosis may be less favorable than CBF AML without such a mutation. When the two subtypes of CBF AML are considered, the majority of reports agree in conferring adverse prognosis in terms of relapse and sometimes [17,27] OS, mainly due to exon 17 mutation, in t(8:21) AML, as documented in some analyses restricted to D816 alteration, compared to other *KIT* mutations [14,26,32,41,46]. On the contrary, the impact of *KIT* mutations on inv(16) is less well concerted (Table 2). Different to t(8;21) AML, most recurrent alterations affect exon 8, in inv(16) AML [19,27,47]. Some authors recognize a correlation between *KIT* mutations and other biological features, such as higher WBCs and circulating blasts at onset [43], assisting the idea that RTK mutations lead to the enhancement of proliferation. Interestingly, the most consistent evidence concerning the unfavorable influence of *KIT* mutations is provided to influence the relapse rate [48,49], rather than the OS, probably owing to the higher sensitiveness to salvage therapy [8]. Schwind et al. [50] investigated the survival implication of non-type A fusion transcripts in inv(16) AML, documenting their association to longer EFS, maybe not strictly depending on the type of transcript, but because of the mutual exclusivity of non-type A and *KIT* mutations. In fact, in patients harboring the most frequent type A fusion transcript, better prognosis is achieved in those with wild-type *KIT*. The divergences among studies could be also related to allele burden, as demonstrated by Allen et al. [15] In the total cohort of CBF AML, outcomes are improved in *KIT*-mutated cases with a higher mutant level of 25%, in terms of relapse risk. Yoon et al. [20] illustrated poorer OS in *CBFB-MYH11* AML with *c-KIT* mutation, by including this genetic alteration in a prognostic-risk scoring, combining age and additional chromosome abnormalities, and attributing an important weight on survival. Moreover, Ishikawa et al. [28] observed that *c-KIT* mutations correlated, statistically, to a lower reduction in fusion transcript levels, after the completion of consolidation chemotherapy, similar to what Qin et al. [47] documented after the first induction, despite neither impacting OS nor RFS. The importance of RTK mutations was related to the emerging possibility to target them by tyrosine kinase inhibitors, based on their activity on different mutations: first-generation TKIs (such as imatinib) work effectively against *KIT* variants of exon 8 and exon 17 mutants involving codon N822, but not against mutants involving codon D816, successfully targeted by other drugs, such as dasatinib and midostaurin [51]. However, the currently available data do not support the routine use of TKIs in association with chemotherapy outside of clinical trials [52]. Moreover, MRD status mainly outweighs the prognostic effects of additional signaling mutations, so that several authors do not take any clinical action based on *KIT* mutation status only. Overall, due to the lack of evidence about its impact on long-term prognosis in AML, the assessment of *KIT* mutational status is not recommended as part of the initial routine diagnostic workup, based on the international ELN recommendations [1].

In second place for incidence, mutations in the *RAS* genes, mainly *NRAS*, are observed in 17–53% of inv(16) AML, i.e., more frequently than in t(8;21) AML [12,15,16,19]. The prognostic role for these RTK mutations has been proposed in only a few studies, such as in the study by Ishikawa et al. [28], suggesting poor RFS for inv(16) patients with *NRAS* mutation. *RAS* variants, belonging to activating mutations, lead to uncontrolled proliferation, as shown by the correlation with higher WBC [15] and may potentially increase sensitivity to chemotherapy [56].

Likewise, *FLT3* is a member of class III tyrosine kinase receptors, and its mutations are relatively rare in CBF AML. While internal tandem duplication (ITD) is the most frequent in cytogenetically normal AML, holding an unfavorable prognostic impact depending on allelic burden, *FLT3* tyrosine kinase domain (TKD) point mutations represent the most common alteration, in inv(16) AML, being associated with BM blast percentage [16]. A clear-cut prognostic significance is not attributable to *FLT3* mutations, because of their negative impact on relapse risk, according to some authors [16,19], whereas Allen et al. [15] showed a favorable role of *FLT3*-TKD high allelic variant on OS.

Remarkably, recent interest is growing about the mutational landscape associated with the rearrangements of the CBF transcriptional complex. Beside the founding mutation, cooperating molecular events contribute to define a distinct gene expression profile of the specific CBF subtype [30]. In inv(16) AML, significantly less co-mutations are detected [25], suggesting that *CBFB-MYH11* promotes leukemogenesis without the need for increasing evolutionary advantage. 

### 2.2. Secondary Additional Chromosomal Abnormalities

Secondary karyotypic aberrations are present in about 40–70% of inv(16) AML, with an incidence increasing with age [10]. In *CBFB-MYH11* AML, most frequent additional chromosome alterations are trisomy of 22 and 21, followed by +8. Similar to other genetic mutations reported above, some authors correlate karyotype abnormalities to clinical manifestations of the disease, such as higher WBC counts at onset [8]. There is consensus about the prognostic favorable role of +22 [6,8] in conferring lower probability of relapse. In contrast, conflicting results had been shown about trisomy 8: some authors documented a negative impact on prognosis [7,19,30], while others reported association with longer survival [23,30]. The number of supplementary chromosome lesions, especially when they are more than 3, in line with other AML subgroups, is associated with a worse outcome [23], according to the prognostic risk scoring by Yoon et al. [20]. Recently, Han et al. [33] have retrospectively drawn up the largest cytogenetic dataset of CBF AML, therefore characterizing and differentiating the genomic features of the two AML subtypes. Trisomies of chromosomes 8, 21 or 22 recurred significantly more frequently in inv(16)-bearing patients, in line with previous studies [6,8,19], such as hyperdiploidy. On the other side, del(9q) and abnormalities in sex chromosomes were more common in t(8;21) AML. The survival analysis of the study revealed different prognostic patterns of cytogenetic factors among the CBF AML subgroups: in inv(16), chromosomal alterations other than +8 were associated with decreased OS, while trisomy 8 was associated with longer survival. Differently, in t(8;21), hypodiploidy was significant for DFS, whereas hyperdiploidy and del(9q) were associated with improved OS [33]. In the current molecular era, these results confirm the timeless relevance of conventional cytogenetic findings on CBF AML prognosis. 

## 3. MRD Monitoring in *CBFB-MYH11* AML

Traditionally, the determinant indicators of prognosis in AML have been identified in the pre-treatment features described above, related to either the patient or the disease features. Another relevant factor impacting on prognosis is represented by the response to cytoreductive therapies, which direct subsequent disease management. Over the last two decades, efforts have been made to improve the assessment of disease response and, especially, the monitoring over time, because although being classified as a favorable risk category, *CBFB-MYH11* AML will experience relapse in nearly 30% of patients, with an estimated RFS rate of 42%, following standard care. The current definition of morphological response lacks sensitivity, and the purpose has been to more accurately detect the residual leukemic burden in BM, far below the 5% blast percentage detectable on microscopic examination [57,58], by detecting what is defined measurable (formerly minimal) residual disease (MRD). Persistent leukemic cells result from cellular resistance mechanisms [59]. Available qualitative molecular tools initially provided partial information about disease status. Firstly, because of low sensitivity techniques, MRD levels below the detection threshold could still be present, despite a negative qualitative reverse transcriptase polymerase chain reaction (RT-PCR) [60,61]. Furthermore, MRD-positive patients will not inevitably be destined to poor outcome, suggesting that monitoring trends of transcript during treatment and further remission phases might be more useful in predicting prognosis [62,63]. As widely applied in other acute and chronic hematologic malignancies, such as acute lymphoblastic or chronic myeloid leukemia [64,65], quantitative molecular methods have become mandatory in longitudinal disease monitoring [66]. Submicroscopic amounts of blast cells could be recognized from the distinct immunophenotypical pattern of lineage-specific antigen expression, identified by multiparametric flow cytometry (MFC), through a combined approach of stable leukemia-associated aberrant immunophenotype (LAIP) and different-from-normal, with a sensitivity of 10^−3^–10^−5^ [67]. Furthermore, with an accuracy up to 10^−6^, quantitative reverse transcriptase PCR (qRT-PCR) is currently considered the gold standard in detecting MRD, applicable in 50–60% of AML patients showing distinct molecular lesions. The ELN MRD working party afforded clinical issues in the application of MRD monitoring in AML by updating recommendations with concepts of complete molecular remission, molecular relapse, and molecular progression [68]. Interestingly, in the same international consensus, MRD status has been recognized to be a better predictor of relapse risk than the presence of cooperating mutations, such as *KIT* and *FLT3-ITD* in CBF AML [1], supporting the essential inclusion of molecular MRD detection into clinical management. Among newer molecular methods, targeted next-generation sequencing (NGS) could provide a complete test of all leukemia-specific genetic aberrations at once, with very high sensitivity. However, NGS data interpretation in MRD monitoring is actually complicated by incurring in some common mutations of any prognostic value but is associated with clonal hematopoiesis of indeterminate potential [69,70]. To note, recent studies have aimed to investigate the clinical relevance of NGS MRD detection, at different timepoints, finding the stronger prognostic impact of NGS MRD status after the first consolidation (2nd timepoint) than at first remission, which could help to identify patient candidates for more aggressive treatment, even when MRD is undetectable by MFC [71]. Indeed, MFC and NGS might be suggested to be used in combination in monitoring the disease, as also MFC and qRT-PCR, especially in the post-induction phase [72,73]. 

The above-mentioned ELN consensus document recommends timepoints and tools for MRD assessment in *CBFB-MYH11* AML. Nevertheless, the lack of technique standardization and heterogeneity of available data results in non-firmly conclusive implications for clinicians, mainly regarding the indication to assign patients to intensive therapeutic approaches. In Table 3, we collected studies in which molecular MRD is found to play a role in impacting outcomes, both in terms of copies of transcript or in terms of logarithmic reduction/rise between different timepoints of detection. 

## 4. ELN Recommendation for MRD Assessment

### 4.1. During the Treatment Phase, We Recommend Molecular MRD Assessment at Diagnosis

In all the studies reported in Table 3, *CBFB-MYH11* fusion transcripts are detected by quantitative molecular methods and normalized to endogenous reference genes. In general, no significant differences in outcomes are observed, depending on the level of transcript at diagnosis [16,75,80,82], based on the great heterogeneity of the fusion gene expression [76]. Marcucci et al. [74], as well as Corbacioglu et al. [81], described only a correlation between copy number and high percentage of BM blasts at disease onset. No correlation was also found with the type of fusion transcript, the type A mutation being the most frequently observed [77,81]. Conversely, Schnittger et al. [77] brought out a strong prognostic impact of the level of transcript on both OS and EFS. A score based on the median expression ratio, after consolidation therapy, and the 75th percentile of the expression ratio at diagnosis was formulated, although it was not possible to identify an absolute threshold to define an early molecular response. However, even if no influence is demonstrated on prognosis, the molecular quantitative assessment of fusion transcript is recommended, at least to evaluate its subsequent modifications.

### 4.2. After Two Cycles of Standard Induction/Consolidation Chemotherapy

The earlier those patients at higher risk of disease relapse are identified, the better diversified therapeutic approaches may be engaged for them. Therefore, several groups have investigated which could be the most relevant timepoint for prognosis during treatment. Some studies proposed a threshold of copy number for discriminating subgroups with shorter remission (i.e., 100 copies in BM [17,73,75], 10 copies in PB [82]). Others, instead, concluded that early assessment of MRD did not predict the disease course [74,77,81], contrary to what is generally thought for early morphologic response, possibly due to the well-known good responsiveness of CBF AML to induction treatment, in addition to a rather slow decline in the disease burden. Interestingly, the prognostic impact of MRD after induction is mostly referred to relapse risk rather than to survival, emphasizing the efficacy of salvage treatment in this category of AML. Of note, Stentoft et al. [78] and Yoon et al. [20] attributed a prognostic relevance not to an absolute level of fusion transcript, but rather to transcript levels, referred to a number of copies at disease onset, namely, a qPCR reduction > or = 3 log, which was associated with longer OS.

Concerning consolidation therapy, the heterogeneity among studies renders highly difficult the comparison among them. In fact, while there is agreement on cytarabine-based consolidation therapy indication [5,78,86,87], controversial questions remain regarding the number of cycles, the most appropriate dose and schedule, as well as the role of combination with other agents. In most studies, two to four cycles have been administered after the attainment of CR and after each cycle MRD was assessed. Early consolidation cycles recur as relevant timepoints. Guièze et al. [80] reported poorer continuous CR (CCR) for values of MRD2 (after first consolidation) > 0.1%; at the same time, the decrease in MRD2 relative to transcript level at diagnosis (deltaMRD2) also strongly impacted the length of remission. Accordingly, the French AML Intergroup [16] demonstrated that a more than 3-log MRD reduction after first consolidation, such as an absolute MRD2 level < or =0.1%, could be used to differentiate low-risk from high-risk patients. Moreover, the threshold of 0.1% resulted to impact on survival also at the end of the second consolidation cycle, as reported by the studies of Duan et al. [31,32] Corbaciouglu et al. [81] underlined the importance of MRD detection in the time window of consolidation and early months after the end of treatment: this timing turns out to be the most informative, in accordance with the median time of relapse occurrence. An early prediction of prognosis allows the clinicians to propose alternative strategies of consolidation. Jourdan et al. [16] recognized in MRD2 the timepoint to evaluate patients for transplant options. Qin et al. [84] recommended allogeneic stem cell transplantation, if *CBFB-MYH11* levels could not decrease to <0.2% after two courses of consolidation, improving RFS and OS [31,32] in these patients at high risk of relapse.

### 4.3. And after the End of Treatment

MRD assessment is recommended at the end of the last consolidation cycle, but its impact on outcome at this timepoint is not unequivocal [60]. If it could be assumed that the majority of long-term survivors maintain PCR-negativity [75,88], it has likewise been reported that a few of the patients in prolonged CR never clear MRD. On the contrary, 10–20% of PCR-negative patients would eventually relapse [89,90,91]. A molecular persistence at low transcript level in BM is common in CBF AML, in the first period after therapy as well as after stem cell transplantation. The possible presence of resistant leukemic clones or quiescent preleukemic cells, potentially responsible for relapse, could be supposed. The latter, called leukemic-initiating cells (LICs) [83] or leukemic stem cells (LSCs) [58,70], are defined as cells capable of initiating disease, self-renewing, with chemo-resistance properties. This subpopulation, usually CD34+/CD38-, may also contribute to subsequent relapse. A combined flow cytometry and fluorescence in situ hybridization examination could help in detecting LSC persistence, as reported by Wang et al. [83], given that the presence of FISH+CD34+CD38- cells predicted OS and RFS. Furthermore, the interpretation of residual rearranged copies is even more difficult because such molecular lesions carried by cells resulting from age-related clonal hematopoiesis of unknown potential [92,93] may not be indicative per se of disease recurrence. One possible explanation lies in biologic mechanisms of a somewhat immunosurveillance effect in preventing disease reappearance, similar to what is documented in the allotransplant setting, that might lead up to the clearance of leukemic compartment, as suggested by the progressive decline of MRD documented, in a timeframe up to 16 months, after consolidation, in some patients without further treatment [62,75,94]. Those findings reinforce the caveat that patients with low molecular burden of disease may need close monitoring, instead of urgent intervention [95]. The recent observation of CBFB-MYH11-specific T cells indicates that CBFB-MYH11 fusion neoantigen is naturally processed and presented on AML blasts and enables T cell recognition and the killing of leukemic cells, supporting the hypothesis of a potential contribution of specific cytotoxic cells in MRD control and CCR maintenance [96].

Hence, unlike other molecular rearrangements, the aim of treatment has to be the transcript reduction below a specific level at definite timepoints, rather than the obtainment of strict MRD negativity [97]. 

### 4.4. During Follow-Up of Patients with PML-RARA, RUNX1-RUNX1T1, CBFB-MYH11, Mutated NPM1, and Other Molecular Markers, We Recommend Molecular MRD Assessment Every 3 Months for 24 Months after the End of Treatment

Once treatments are completed, for patients not candidate to further therapy lines, it will be necessary to set up, on a medium-long-term basis, a reliable follow-up in tracking either an ongoing response or, promptly, an impeding relapse. Even at this stage, a consensus in monitoring MRD kinetics emerges from published studies. In fact, relapse could effectively be predicted by a comparison between longitudinal sampling, rather than by overcoming a definite threshold. ELN updated recommendations reflect such an awareness: molecular relapse, as well as molecular progression, is defined by a logarithmic increase in MRD levels between two consecutive samples, underlining the dynamic interpretation of a molecular parameter. In addition, this approach makes data more comparable, despite methodological and clinical differences among studies. The use of a logarithmic increase for defining relapse and progression is therefore operational in CBF AML, because of the predominantly slow pattern of regrowth, depending on biological factors, which allows one to observe evolutive trends before morphological relapse. Some concordance exists in the proposed patterns of molecular values: as a reduction of 2–3 log after induction/consolidation [75,78] is expected, a controlled disease is not supposed to show a 10-fold increasing of MRD, during follow-up. A monitoring schedule is pivotal for avoiding the missing herald of relapse, so that, at this point, a relevant interrogative matter is about frequency in sampling. International guidelines indicate to assess MRD every 3 months for at least the first 2 years after the end of treatments, because relapse occurs after 2 years of complete remission, more rarely [79,81,85]. This timeframe has been consistently derived from several studies that described that in patients monitored at 3-month intervals a clinical recurrence was predictable by rising MRD [70,77,80,83]. Krauter et al. [76], such as other authors earlier [74], recognized two patterns of relapse: some patients obtained molecular negativity directly after induction/consolidation, with a secondary transcript level increase, whereas others maintained a detectable disease at the end of treatment, but, in both instances, the median interval between increasing MRD and hematological relapse was beyond 3 months. When the kinetics of transcript levels rising was evaluated, a slower rate of MRD increasing for *CBFB-MYH11* AML was observed, as compared to *NPM1* or *PML-RARA* positive AML, with a BM doubling time of 36 days and the longest lag before morphological relapse (even 8 months) [98], suggesting the need for longer monitoring. However, these considerations may be challenged because the possibility of more rapid relapse kinetics cannot be excluded [79]. Relevant to this, Yin et al. [82] reported a median increment in transcript levels of about 0.5 log_10_/month, so that a 3-monthly assessment schedule might fail to identify a potential MRD increase up to 3 logs, impairing potential pre-emptive therapeutic strategies, including SCT [99]. Puckrin et al. [85] emphasized that the monitoring approach indicated by ELN could fail to detect, in a timely manner, relapsing patients, because the majority of clinical recurrences occurred within 100 days from molecular relapse. Reasons could be attributed to real-life limitations, such as difficulties in fulfilling sampling intervals, differences in either specimen quality or sensitivity of available molecular assays. This evidence warrants consideration about the informative value of MRD, in terms of treatment decision: if, on the one hand, clear recommendations have been formulated about timeframes of monitoring, no formal instructions are available to indicate either therapy changes or pre-emptive interventions, leaving the precautionary management of impending relapse at the single center’s discretion. 

### 4.5. In BM and in PB. Alternatively, PB May Be Assessed Every 4–6 Weeks

Whether PB sampling can definitely succeed in replacing bone marrow sampling for MRD testing remains an open point. Sensitive and reliable blood-based assays would be an attractive possibility, given the less invasive nature compared to BM aspirations. The two sources have been studied as alternatives in several studies, albeit the most clinically relevant findings are derived from BM specimens. As detailed in Table 3, some authors, in the identification of significant thresholds of fusion transcripts for prognosis, included PB cut-offs about five times lower than in BM, taking into consideration the difference in sensitivity. This interest has grown from evidence in other AML subtypes, namely, *NPM1*-mutated AML, in which discrimination for survival was better by using PB rather than BM [100]. Boeckx et al. [101] reported preliminary data in support of a moderate correlation in PB-BM pairs, although generally higher levels are found in BM, suggesting that PM examinations could be performed every 2–3 months during follow-up, with further BM aspirations considered to be necessary only in the case of rising transcripts in PB. Encouraging results also emerged from Stentoft et al. [78], who demonstrated a convincing correlation between PB and BM, and from Ommen et al. [98], who provided preliminary data suggestive of equal usefulness of either BM or PB sources. Moreover, Guièze et al. [80] revealed a high degree of concordance for levels of MRD higher than 0.1%, whereas for lower levels, BM appeared more sensitive. Interestingly, Corbacioglu et al. [81] recommended to use BM samples during consolidation therapy, while, during follow-up, MRD could be measured on PB for evaluation of longitudinal rising levels during CCR. The UK MRC trial group [82] offered an optimal schedule for molecular monitoring and confirmed that both BM and PB were comparable for MRD detection after the end of treatment, even if in 10–15% of patients, negative PB showed discordance with MRD positivity detection in BM. 

Recently, Skou et al. [99] reinforced the notion that an effective surveillance of imminent relapse could be achieved through frequent PB sampling. In fact, both the persistent molecular positivity in BM, despite continuous remission, and the relative absence of progenitors in peripheral blood should mean higher predictivity in detecting MRD in PB. A positive molecular finding from peripheral blood is more suggestive of imminent relapse. Doubts might arise about whether PB monitoring provides sufficient lead time to prevent clinical relapse. Despite earlier occurrence, no meaningful difference arose from comparisons between the rate of leukemic regrowth between PB and BM. In addition, it is possible to collect samples from peripheral blood with higher frequency [53] (possibly monthly, during the first year following the completion of therapy), which, in turn, are easily accessible and more acceptable by patients, outweighing the gap of molecular load about 0.5–1 log_10_ between BM and PB. Furthermore, additional information from PB with respect to BM could be provided, by gene expression assays and combined multiparameter flow cytometry, able to detect with more specificity, even though with less sensitivity, potential circulating leukemic cells, due to the background of less progenitor populations compared to bone marrow [58].

Hence, not only a comparable but also a better disease surveillance could be assured by monthly PB sampling, which allowed us to efficiently identify molecular relapse, as an increase by 1 log_10_ between two positive samples, confirmed by paired BM and PM assay, after 4 weeks [99]. 

### 4.6. MRD Should Be Assessed Pre Transplant. MRD Should Be Performed Post Transplant

Regarding the transplantation setting, although assessment is recommended before and after procedure, no specific indication is provided about the interpretation of MRD values. In consideration of the relatively satisfying long-term control of disease with repeated high or intermediate-dose cytarabine consolidation or alternatively autologous stem cell transplantation (auto-SCT), CBF AML are not usually candidated for allo-SCT in first CR, contemplating this procedure for patients in second remission [102], although data about post-remission therapy still remain debated. As reported in some studies [103,104,105], in first remission no differences have been found in terms of LFS and RI between auto-SCT and allo-SCT, to the detriment of the higher TRM (therapy-related mortality) of the non-autologous approach. On the other hand, some authors [20,106,107], comparing non-allogeneic and allo-SCT consolidation treatments, underlined favorable OS for the latter, encouraging the use of frontline transplantation for subgroups of CBF AML with adverse risk characteristics, including cases showing MRD positivity. The negative impact on the survival of residual disease prior to allogeneic SCT is demonstrated by several studies, as showed in a meta-analysis of Buckley et al. [108]. In case of indication of allogeneic transplantation in MRD-positive patients, haploidentical allograft seems to be superior to match sibling donor, suggesting strong anti-leukemia effects in eradicating pre-SCT residual disease [109,110,111]. Moreover, in this peri-transplant setting, rather than single timepoint positive or negative status, a significant role is attributed to the MRD dynamic trend among prior chemotherapy cycles. Interestingly, Qin et al. [84] identified as a sole independent adverse prognostic factor for CIR, DFS and OS a transcript level reduction less than 3 log after course 2 consolidation, and only in this poor MRD category of patients allo-SCT could significantly improve outcome, without any advantage for good MRD patients. When MRD trend after second consolidation indicates an SCT approach, several studies stress the negative impact of detectable MRD on the risk of post-transplant relapse [112,113], and achieving MRD negativity might result in improved transplantation outcome. However, it remains unclear whether patients with positive MRD should be straightly directed to SCT or should receive further chemotherapy. In fact, precisely in poor MRD responders, transplantation by exploiting intensive conditioning regimens, rather than reduced-intensity schemes, and utilizing alternative or mismatched donors, theoretically eliciting stronger GvL [114], could offer an advantage on outcome. Interestingly, among a series of 58 unselected AML patients receiving SCT, Zhang et al. [115] proved transplantation as a safe choice also for the treatment of refractory/relapsed (r/r) cases. In details, while the 5-year OS of r/r AML patients was 54.21% lower that documented in non-r/r patients (71.82%), the 5-year EFS was not statistically different between the two groups (53.54% versus 62.07%). Of note, the 5-year OS rates of r/r AML patients who had subsequently obtained CR and those with persistent disease before SCT were not different, 56.06% and 51.85%, respectively (*p* = 0.6408), due to the rapid and early tapering of immunosuppression therapy after transplantation and prophylactic donor lymphocyte infusion.

To reiterate the relative significance of positive MRD pre SCT, Zhao et al. [109] reported that instead of MRD before SCT, the unfavorable effect on prognosis in multivariate analysis is attributed to detectable MRD post haploidentical SCT, without the necessity of further intensive chemotherapy for MRD-positive patients prior to transplantation. Yalniz et al. [95] showed that there is no impact of the MRD level by qRT-PCR on the relapse incidence, even in the patients with the highest disease burden. In addition, in this study, two MRD checkpoints post SCT were identified: the presence of MRD on day +30 did not indicate impending relapse, whereas patients who had detectable disease on day +100 had a 3-year relapse incidence of 27.6% versus 9.7% for patients without residual disease, although not reaching statistical significance. Monitoring MRD after the first 3 months of transplantation rather than before could be more informative about the risk of relapse: lower LFS and higher CIR resulted for a decrease of less than 3 logs compared to pre-treatment levels [116]. Pre-emptive therapeutic strategies, including approaches selectively increasing GvL (graft versus leukemia), to target MRD persistence post allogeneic SCT in AML patients with inv(16)/t(16;16) are warranted.

## 5. Novel Therapies

In recent years, the approval of several novel agents for treating AML was obtained; the most remarkable for improving outcomes in inv(16) is the addition of the anti-CD33 monoclonal antibody gemtuzumab ozogamicin (GO) to the remission induction cycle [117,118,119]. To note, data have been published about the first steps toward specific T-cell immunotherapy in fusion gene-driven AML [96]. *CBFB-MYH11* protein could act as neoantigen, giving rise to the potential development of a personalized adoptive TCR T-cell strategy. Because of the early and essential role in leukemogenesis and the specificity persistence in blast cells, *CBFB-MYH11* should represent an optimal target, with minimal risk of off-tumor toxicity. Recent translational studies are providing the basis for future targeted therapeutic approaches. For example, advances in treatment would be warranted focusing on the molecular processes involved in leukemogenesis driven by fusion protein CBFB-SMMHC encoded by the *CBFB-MYH11* founder gene, by the potential target of specific or indirect inhibitor [120], or exploring the disease mechanism of oncogene-induced chromatin remodeling [121]. 

## 6. Conclusions

In recent years, the advances in molecular diagnostic and monitoring, with the simultaneous incoming of new therapeutic agents, have led to significant improvements in clinical AML management. Awada et al. [122] recently integrated cytogenetic and gene sequencing data from a multicenter cohort of nearly 7000 AML patients that were analyzed using standard and machine learning methods to generate a novel AML molecular subclassification with biological correlates corresponding to underlying pathogenesis. Despite the heterogeneity of AML genomics, non-random genomic relationships were capable of identifying four novel unique genomic clusters with a distinct prognosis, regardless of the availability of pathomorphological or anamnestic information. MRD monitoring actually supersedes other well-recognized clinical features, with independent prognostic value, at least in some AML subgroups. European Leukemia Net has offered recommendations about quantitative and qualitative MRD monitoring, as part of the standard of care for AML patients. MRD threshold levels might prelude worse outcome, as clinical relapse could be accurately predicted by sequential sampling during follow-up in both BM and PB. Increasing MRD value or molecular relapse ensures a window of opportunity to adapt risk-directed interventions before overt progression. In CBF AML, the MRD-negative groups displayed more favorable RFS than those with MRD positivity, and OS was also superior in the MRD-negative group. Moreover, the CIR was statistically significantly lower in the MRD-negative group, when considering the most significant cut-off MRD level of 0.1% [123]. Nevertheless, the standardization of molecular tools, including the application of newer technologies, and timepoints of MRD investigations in CBF AML, in order to guide therapeutic decisions, is still controversial. It is argued whether patients in CR1 should be offered transplant, based only on early response, by considering an estimated transplant-related mortality of 10% to 15%, while at least half of these patients would not eventually relapse. Therefore, it is not actually confirmed that a pre-emptive approach would be beneficial, when considering the slower kinetics of leukemic growth, the real-life MRD monitoring limitations and the overall good responses achievable with salvage therapy, in case of a full-blown relapse. 

Ultimately, further studies are needed to improve knowledge about the best employment of MRD information to improve the clinical outcomes of CBF AML patients. 

## Figures and Tables

**Table 1:** Prognostic impact of clinical features at diagnosis in patients with AML showing inv(16)/t(16;16).

Reference; Design of Study	N. of Patients with inv(16)	Median Age, Years (Range); Median Follow-Up, Months	Survival Outcome	Factors of Prognostic Relevance	
				Patient’s Characteristics and Clinical Features	Genetic Features (Other than *KIT* Mutations)
Delaunay et al. [10], Blood 2003 retrospective	110	34 (0.7–64) 68.4	CR 93% 3-y OS 58% 3-y DFS 48% 3-y CIR 42%	UVA—WBC > 120 × 10^9^/L: lower CR PLT < 30 × 10^9^/L: lower CR, OS in CR Age >35: lower 3-y DFS, OS in CR; higher CIR MVA—WBC > 120 × 10^9^/L: lower CR PLT < 30 × 10^9^/L lower OS in CR Age > 35: lower 3-y DFS, OS in CR	+22: lower CR
Schlenk et al. [8], J. Clin. Oncol. 2004 prospective	201	42 (17–60) 36	CR 89% CR2 78% 3-y OS 74% 3-y RFS 58%	Older age, higher WBC: increased early/hypoplastic death	+22: higher RFS
Marcucci et al. [6], J. Clin. Oncol. 2005 prospective	168	40 (17–77) 76.8	CR 87% 5-y OS 54% 5-y CIR 57%	Lower PLT, hepatomegaly: lower CR Older age, lower PLT: lower OS Older age: lower OS after relapse In younger than 60, sex (male): lower CIR	+22: lower RR In younger than 60, secondary chromosome abnormalities: lower CIR
Boissel et al. [12], Leukemia 2006; retrospective	47	33 (1–75) * 52.8 *	CR 89% 6-y OS 71% 6-y EFS 60%	NA	*FLT3*mut: lower CR, OS, EFS *
Appelbaum et al. [7], Br. J. Haematol. 2006; retrospective	196	41 (16–83) 108	CR 85% 5-y OS 50% 5-y RFS 44%	UVA—Older age, secondary AML: lower CR * UVA—Older age, PB and BM blast %: lower OS * MVA/UVA—Older age, PB blast%: lower RFS *	−7/7q-: higher RD * +8, complex abnormality: lower OS *
Wang et al. [13], Biochem. Biophys. Res. Commun. 2012	11	28 (16–64)	CR 81.8%	WBC >100 × 10^9^: lower CR and OS *	NA
Kim et al. [14], Ann. Hematol. 2013 retrospective	39	38 (18–69) 27	CR 100% 2-y OS 57.1% 2-y EFS 47.5%	MVA—Older age: lower EFS	NA
Allen et al. [15], Leukemia 2013 retrospective	155	39 (15–70) * 99.6 *	10-y OS 54%	NA	*FLT3-*TKD^HIGH^: lower RR, higher OS *FLT3-*ITD^HIGH^: higher RR, lower OS * CBL^HIGH^: higher OS *
Jourdan et al. [16], Blood 2013 prospective	102	42 (18–60) 32	3-y RFS 61% 3-y CIR 34% 3-y OS 86%	UVA—Higher WBC: higher SHR * Older age, BM blast %: lower OS * MVA—3-log MRD2 reduction or MRD2 < 0.1%: lower CIR, higher RFS, higher OS from CR *	RTKmut: higher CIR, lower RFS *
Hoyos et al. [17], Eur. J. Haematol. 2013 prospective	76	42 (18–68) 55	CR 84% 5-y CIR 29% 5-y DFS 58% 5-y OS 64%	Age > 50: lower CR, lower OS* WBC > 20 × 10^9^/L: higher CIR, lower DFS, lower OS * High copies at diagnosis: higher CIR, lower DFS, lower OS * High MRD after induction: higher CIR, lower DFS, lower OS High MRD after consolidation: higher CIR, lower DFS, lower OS	*BAALC* and *MN1* overexpression: higher CIR, lower DFS
Cairoli et al. [18], Am. J. Hematol. 2013 prospective	58	42 (15–60) 50	CR 96.5%; CR2 74% 5-y RI 48.4% 5-y OS 69.2%	UVA—Age > 43: lower OS MVA—Age > 43: lower OS Higher WBC: higher RI	NA
Paschka et al. [19], Blood 2013 prospective	176	41 (18–74) 72.4	CR 90% 6-y RFS 52% 6-y OS 66%	MVA—Higher WBC: lower RFS Older age: lower OS	UVA—+22: higher RFS +8, *FLT3* mutation: lower OS MVA—+8, *FLT3* mutation: lower OS
Yoon et al. [20], Bone Marrow Transplant. 2014 retrospective	71	39 (18–89) * 61.8 *	NA	UVA—Age > 40: lower OS, higher CIR * Post-induction MRD reduction < or =3-log: lower OS * MRD after final treatment undetectable: higher OS	NK mosaicism: higher OS, higher EFS ^§^ UVA—additional chromosome > or =2: lower OS, higher CIR *
Jung et al. [21], Anticancer Res. 2014 retrospective	16	47 (18–75) NA	CR 92.3% * Median OS 80.6 months * Median RFS 68.4 months *	PLT < 20 × 10^9^/L, PB blasts > 50%, BM blasts > 50%: lower OS * PLT < 20 × 10^9^/L, BM blasts >50%: lower LFS *	Y deletion: higher OS and LFS *
Brunner et al. [22], Leuk. Res. 2014 retrospective	320	54 (15–84) * NA	1-y OS 71.9% 3-y OS 57.3% 5-y OS 46.9%	Older age: higher early death rate, lower OS * Black ethnicity, year of diagnosis before 2003: lower OS *	NA
Mosna et al. [23], Am. J. Hematol. 2015 retrospective	112	45.1 (15–73) 73.4	CR 93.8% ^§^ 5-y OS 67% * 10-y OS 63.9% * 5-y DFS 58.2% * 10-y DFS 54.8% * 5-y EFS 53.9% * 10-y EFS 49.9% *	UVA—PLT ≤ 20 × 10^3^/mm^3^, failure to achieve CR1 after induction therapy: lower OS MVA—Age > 60, PLT ≤ 20 × 10^3^/mm^3^: lower OS	+22, +8: higher OS and DFS * Additional cytogenetic abnormalities > or =3: lower DFS, EFS and OS *
Yui et al. [24], Ann. Hematol. 2017 retrospective	28	45 (15–80) * NA	3-y RFS 48.6% * 3-y OS 69.9% * 3-y CIR 46.7% *	Age > 60, no HDAC as post-remission therapy: lower OS and RFS *	NA
Prabahran et al. [25], Eur. J. Haematol. 2018 retrospective	30	46.5 (17–73) 31.4	CR 97% * 5-y OS 71% * 5-y RFS 39% ^§^ 5-y RR 57% ^§^	UVA and MVA—age > 50: lower OS * UVA—WBC > 40 × 10^9^/L: lower RFS *	RTKmut: no impact on OS, RFS
Shin et al. [26], Ann. Hematol. 2019 retrospective	111	45 (17–85) NA	3-y EFS 47.1% 3-y OS 59.9%	UVA—Age > 60, number of induction cht > 1, not CR after first induction, not CR before SCT: lower OS MVA—not CR before SCT: lower OS	del(7q): higher OS (NS)
Opatz et al. [27], Leukemia 2020	162	44 (17–83) 43.2	CR 97.1%	UVA—Age > 60: lower OS	UVA—+8, +22: higher OS (NS)
Ishikawa et al. [28], Blood Adv. 2020 prospective	67	37 (17–64) 52.2	2-y RFS 59.6%	MVA—MRD ≥ 50 copies/μg RNA after 3 courses of consolidation: lower RFS	MVA—Loss of X/Y, *NRAS* mutation: lower RFS
Ustun et al. [29], Int. J. Lab. Hematol. 2020 retrospective	290	49 (5–78) NA	Median EFS 25.5 m Median DFS 29.5 m	MVA—Age > or =43: lower EFS, DFS, OS WBC ≥ 98 × 10^9^/L: lower EFS, DFS	NA
Jahn et al. [30], Blood Adv. 2020 prospective	160	46 (18–77) 51.6	CR 92%	Age: lower OS WBC, t-AML: lower RFS	+8, *FLT3*-ITD, *TET2*, *DNMT3A*: lower OS * WT1wt: higher OS * NRASwt: higher OS (NS) *
Duan et al. [31], Br. J. Haematol. 2021 retrospective	58	38 (17–66) 29.8	CR 98.3% 3-y CIR 29.4% 3-y CIM 24.4%	Age > 41: lower RFS MRD > 0.1% after 2 courses of consolidation: lower RFS, EFS	NA
Duan et al. [32], Ann. Hematol. 2021 retrospective	68	39 (15–70) * 26 *	CR 99.5% * 3-y CIR 29.4% * 3-y CIM 27% *	MRD < 0.1% after 2 course of consolidation: higher RFS, OS	NA
Han et al. [33], Blood Adv. 2021 retrospective	290	50 (5–81) 39.6	CR 93% 5-y OS 68% 5-y DFS 47%	UVA—Age: lower OS, DFS	UVA—Hyperdiploidy, +8, secondary chromosomal abnormalities: higher DFS MVA—Chromosomal abnormalities other than +8: lower OS +8: higher OS

CR: complete remission after induction; CR1: 1st CR; CR2: 2nd CR; OS: overall survival; RFS: relapse-free survival; EFS: event-free survival; DFS: disease-free survival; LFS: leukemia-free survival; CIR: cumulative incidence of relapse; CIM: cumulative incidence of mortality; RI: relapse incidence; RR: relapse rate; RD: resistant disease; cht: chemotherapy cycle; HDAC: high-dose cytarabine; mut: mutated; wt: wild-type; UVA: univariate analysis; MVA: multivariate analysis; WBC: white blood cell count; PLT: platelets count; PB: peripheral blood; BM: bone marrow; MRD: measurable residual disease; MRD2: MRD before second consolidation course; SCT: stem cell transplant; SHR: specific hazard of relapse; AML: acute myeloid leukemia; t-AML: therapy-related AML; NK normal karyotype; NA: not available data; NS: not statistically significant data; * Data referred to entire cohort of the study; ^§^ Data referred to *CBFB-MYH11* AML cohort.

**Table 2:** Prognostic relevance of *KIT* gene mutation in AML positive for inv(16)/t(16;16).

Reference	N.	Median Age, Years (Range)	*KIT* Exons Analyzed	Proportion of Patients with *KIT* Mutations, %	Prognostic Relevance of *KIT* Mutations
Care et al. [48], Br. J. Haematol. 2003	63	43.9 (15–74)	8, 17	32 (20/63)	Higher RR with *KIT* exon 8 mutation
Boissel et al. [12], Leukemia 2006	47	33 (1–75) *	8, 17	22 (10/46)	No impact on OS, RFS, EFS In t(8;21): negative impact on OS, RFS, EFS and association to higher WBC
Cairoli et al. [41], Blood 2006	25	51 (17–88)	8, 11, 17	48 (12/25)	No impact on RI, OS In t(8;21): negative impact on OS, RFS, EFS for D816 and association to higher WBC and extramedullary leukemia
Paschka et al. [43], J. Clin. Oncol. 2006	61	NA	8, 17	29.5 (18/61)	Higher CIR in mut*KIT* patients, mainly in exon 17 mutations (six times RR) Inferior OS in MVA in mut*KIT* patients Association to higher PB blast percentage and older age
Marková et al. [53], Leuk. Lymphoma 2009	26	29.3 (1.6–72.2) *	8, 9, 10, 11, 17, 18	50 (13/26)	No impact on RFS, OS
Park et al. [49], Leuk. Res. 2011	38	NA	8, 17	34 (13/38)	Lower CR with *KIT* exon 8 mutation; no impact on EFS, OS In t(8;21): negative impact on OS, EFS with *KIT* exon 17 mutation
Wang et al. [13], Biochem. Biophys. Res. Commun. 2012	11	28 (16–64)	8, 17	28.9 (22/76) *	Lower CR with *KIT* exon 17 mutation * Lower OS, RFS in mut*KIT* patients *
Huh et al. [46], Am. J. Hematol. 2012	35	41 (15–75) *	8, 10, 11, 12, 13, 17	23 (21/91) *	No impact on OS In t(8;21): negative impact on OS, EFS, LFS with *KIT* exon 17 mutation (D816)
Kim et al. [14], Ann. Hematol. 2013	39	38 (18–69)	8, 10, 11, 12, 13, 17	26.4 (32/121) *	No impact on OS, EFS In t(8;21): negative impact on OS, EFS with *KIT* exon 17 mutation (D816)
Allen et al. [15], Leukemia 2013	155	39 (15–70) *	8,9, 10, 11, 17, 18	35 (54/155)	No impact on CIR, OS in MVA In t(8;21): negative impact on CIR for *KIT*^HIGH^ mutant level
Jourdan et al. [16], Blood 2013	102	42 (18–60)	8, 17	18 (18/102)	Negative impact on CIR (*p* = 0.057) *
Hoyos et al. [17], Eur. J. Haematol. 2013	76	42 (18–68)	8, 17	49 (19/39)	No impact on CIR, DFS, OS In t(8;21): negative impact on CIR
Cairoli et al. [18], Am. J. Hematol. 2013	58	42 (15–60) 50	2, 8, 10, 11, 17	25.9 (15/58)	No impact on CR, RI, OS
Paschka et al. [19], Blood 2013	176	41 (18–74)	8, 10, 11, 17	37 (65/175)	Lower RFS with *KIT* exon 8 mutation Association to higher WBC and PB blast %
Riera et al. [54], Oncol. Rep. 2013	14	42.7 (19–64) *	8, 9, 10, 11, 13, 14, 17	28.6 (4/14)	No impact on CR, OS, DFS Association to higher lactate dehydrogenase level
Schwind et al. [50], Blood 2013	208	41 (17–74)	8, 17	24 (48/208)	Lower OS, EFS
Yoon et al. [20], Bone Marrow Transplant. 2014	71	39 (18–89) *	17	25 (6/24)	Lower OS
Park et al. [55], Ann. Lab. Med. 2015	21	47 (16–82)	8, 17	14.3 (3/21)	No impact on DFS, OS In t(8;21): negative impact on DFS, OS
Qin et al. [47], Leuk. Res. 2014	98	(0.5–73)	8, 17	29.6 (29/98)	No impact on CIR, DFS, OS Less reduction in fusion transcript levels after first induction therapy In t(8;21): negative impact on OS, DFS, CIR
Mosna et al. [23], Am. J. Hematol. 2015	112	45.1 (15–73)	8	10.2 (4/39)	No impact on OS In t(8;21): negative impact on OS
Yui et al. [24], Ann. Hematol. 2017	28	45 (15–80) *	8, 17	16 (10/28)	Lower OS, RFS with *KIT* exon 17 mutation (D816)
Prabahran et al. [25], Eur. J. Haematol. 2018	30	46.5 (17–73)	NA	58 (7/12)	No impact on OS, RFS
Shin et al. [26], Ann. Hematol. 2019	111	45 (17–85)	17	NA	No impact on OS, EFS In t(8;21): negative impact on OS, EFS with *KIT* exon 17 mutation (D816)
Opatz et al. [27], Leukemia 2020	162	44 (17–83)	17	26 (41/162)	No impact on RFS, OS In t(8;21): negative impact on RFS with *KIT* exon 17 mutation (D816)
Ishikawa et al. [28], Blood Adv. 2020	67	37 (17–64)	8, 10, 11, 17	31.3 (21/67)	No impact on OS, RFS In t(8;21): negative impact on OS, RFS with *KIT* exon 17 mutation; association to higher WBC and BM blast %; association to MRD level after consolidation
Jahn et al. [30], Blood Adv. 2020	160	46 (18–77)	8, 17	26	In t(8;21): negative impact on OS with *KIT* exon 17 mutation
Duan et al. [31], Br. J. Haematol. 2021	58	38 (17–66)	8, 17	27.5 (16/58)	Lower RFS
Duan et al. [32], Ann. Hematol. 2021	68	39 (15–70) *	8, 17	27.9 (19/68)	Lower RFS, OS with *KIT* mutation, especially with *KIT* exon 17 mutation (D816, D820) *
Han et al. [33], Blood Adv. 2021 retrospective	290	50 (5–81)	17	13	In t(8;21): negative impact on OS, DFS with *KIT* exon 17 (D816) mutation

CR: complete remission after induction; RFS: relapse-free survival; OS: overall survival; RI: relapse incidence; RR: relapse rate; DFS: disease-free survival; EFS: event-free survival; LFS: leukemia-free survival; CIR: cumulative incidence of relapse; MVA: multivariate analysis; MRD: measurable residual disease; WBC: white blood cell count; PB: peripheral blood; BM: bone marrow; NA: not available data. * Data referred to entire cohort of the study.

**Table 3:** Molecular MRD monitoring in AML with *CBFB-MYH11* fusion transcript.

Reference	N. of Patients; Median Age, Years	Median Follow-Up, Months; Outcomes	Timepoint	PB or BM	Prognostic Transcript Level Cutoff or Trend of MRD Dynamics	Associated Risk	Sensitivity of the Assay
Marcucci et al. [74], Leukemia 2001	16 NA	NA CR 100%	At the end of treatment	BM	*CBFB-MYH11*/18S × 10^6^ >10 copies	Shorter CR duration and higher risk of relapse for *CBFB-MYH11*/18S × 10^6^ >10 copies	10^−4^
Buonamici et al. [61], Blood 2002	2149	51 CR1 72% 3-y DFS 63% 3-y OS 82%	Any time during CR	PB/BM	*CBFB-MYH11/ABL* < 0.12% *CBFB-MYH11/ABL* > 0.25%	High probability of durable remission for *CBFB-MYH11/ABL* < 0.12% High risk of relapse for *CBFB-MYH11/ABL* > 0.25%	10^−5^
Guerrasio et al. [75], Leukemia 2002	36 35	27.5 NA	After induction	BM	*CBFB-MYH11/ABL* × 10^4^ > 100 copies	High risk of relapse for *CBFB-MYH11/ABL* × 10^4^ >100 copies	10^−5^
			After consolidation	BM	*CBFB-MYH11/ABL* × 10^4^ > 10 copies	High risk of relapse for *CBFB-MYH11/ABL* × 10^4^ >10 copies	
			At any time during CR	BM	*CBFB-MYH11/ABL* × 10^4^ < 1 copy	Higher probability of CCR	
Krauter et al. [76], J. Clin. Oncol. 2003	15 ^§^ 39 *	19 * NA	At least at one time point after induction	BM	*CBFB-MYH11*:GAPDH in CR/*CBFB-MYH11*:GAPDH at diagnosis > or = 1%	Shorter RFS *	10^−5^
Schnittger et al. [77], Blood 2003	122 ^§^ 48.9 ^§^	17.7 ^§^ NA	At diagnosis AND after consolidation	BM	*CBFB-MYH11/ABL* × 10^2^ < 75th percentile at diagnosis AND *CBFB-MYH11/ABL* < 0.014 after consolidation	2-y OS 100% (vs. 69% if initial level >75th percentile and/or more than 0.014 after consolidation) ^§^ 2-y EFS 100% (vs. 40% if initial level > 75th percentile and/or more than 0.014 after consolidation) ^§^	10^−5^
Perea et al. [73], Leukemia 2006	35 ^§^ 43 ^§^	34 * 2-y LFS 50% ^§^ 2-y OS 64% ^§^ CR 84% *	After induction	BM	*CBFB-MYH11/ABL* × 10^4^ < or = 100 copies	2-y CIR 35% (vs. 58% >100 copies) * (NS)	10^−5^
			After intensification	BM	*CBFB-MYH11/ABL* × 10^4^ < or = 10 copies	2-y CIR 36% (vs. 70% >10 copies) * (NS)	
			At the end of treatment	BM	*CBFB-MYH11/ABL* × 10^4^ < or = 10 copies	2-y CIR 26% (vs. 100% >10 copies) ^§^	
			At follow-up	BM	*CBFB-MYH11/ABL* × 10^4^ < or = 10 copies	2-y CIR 13% (vs. 78% >10 copies) *	
Stentoft et al. [78], Leuk. Res. 2006	13 ^§^ 39 *	NA	After induction	PB/BM	<2-log reduction of the fusion transcript level	Shorter EFS (*p* < 0.014) *	10^−4^
Lane et al. [79], Leuk. Lymphoma 2008	17 ^§^ 35 ^§^	34 * NA	At follow-up	BM	> or = 1-log rise in transcript levels in consecutive samples in CR	Predictive for imminent morphological relapse and shorter LFS (*p* = 0.008) *	10^−6^
Guièze et al. [80], Leukemia 2010	59 36	26.5 2-y CCR 63% 2-y OS 88%	At CR achievement	PB/BM	*CBFB-MYH11/ABL* < 0.5%	2-y CCR 76% (vs. 36% > 0.5%)	NA
			After 1st consolidation (MRD2)	PB/BM	*CBFB-MYH11/ABL* < 0.1%	2-y CCR 74% (vs. 40% > 0.1%)	
				PB/BM	MRD2 transcript level/ *CBFB-MYH11* transcript level at diagnosis (deltaMRD2) decrease >3 log	2-y CCR 83% (vs. 28% if deltaMRD2 decrease < 3 log) 2-y OS 100% (vs. 67% if deltaMRD2 decrease < 3 log)	
			At the end of consolidation	PB	*CBFB-MYH11/ABL* undetectable	2-y CCR 85% (vs. 13% for detectable MRD)	
Corbacioglu et al. [81], J. Clin. Oncol. 2010	52 NA	47 NA	From 1st consolidation until up to 4 weeks after last consolidation (checkpoint I)	BM	At least 1 PCR negative sample	2-y RFS 79% (vs. 54% for patients who never achieved PCR negativity during consolidation)	10^−4^
			From 1st consolidation until up to 3 months after last consolidation (checkpoint II)	PB/BM	At least 2 PCR negative samples	2-y RFS 91% Longer OS	
			At follow-up	BM	Conversion of PCR negativity to positivity (>10 copies/B2M × 10^6^)	High risk of relapse	
Yin et al. [82], Blood 2012	115 ^§^ 38 ^§^	36 * CR 92% ^§^ 5-y CIR 23% ^§^	At remission after induction	PB	*CBFB-MYH11/ABL* × 10^5^ < 10 copies	5-y CIR 21% (vs. 56% 10–500 copies) ^§^ 5-y survival after CR 89% (vs. 45% 10–500 copies) ^§^	10^−5^
			After courses 3 and 4	PB	*CBFB-MYH11/ABL* × 10^5^ < 10 copies	5-y CIR 36% (vs. 78% > 10 copies) ^§^	
			At follow-up (4 weeks after last treatment)	PB	*CBFB-MYH11/ABL* × 10^5^ < 10 copies	5-y EoR 7% (vs. 97% > 10 copies) ^§^ 5-y EoS 91% (vs. 57% > 10 copies) ^§^	
				BM	*CBFB-MYH11/ABL* × 10^5^ < 50 copies	5-y EoR 10% (vs. 100% > 50 copies) ^§^ 5-y EoS 100% (vs. 25% > 50 copies) ^§^	
Jourdan et al. [16], Blood 2013	102 ^§^ 42 ^§^	32 * 3-y RFS 61% ^§^ 3-y CIR 34% ^§^ 3-y OS 86% ^§^	Before 2nd consolidation (MRD2)	BM	> or = 3-log MRD2 reduction	3-y CIR 22% (vs. 54% for patients who did not achieve a 3-log MRD2 reduction) * 3-y RFS 73% (vs. 44% for patients who did not achieve a 3-log MRD2 reduction) * 3-y OS 90% (vs. 71% for patients who did not achieve a 3-log MRD2 reduction) (NS) *	NA
					MRD2 < or = 0.1%	Lower CIR and longer RFS	
Hoyos et al. [17], Eur. J. Haematol. 2013	76 ^§^ 42 ^§^	55 * CR 84% ^§^ 5-y CIR 29% ^§^ 5-y DFS 58% ^§^ 5-y OS 64% ^§^	After induction	BM	*CBFB-MYH11/ABL* < 100 copies	DFS 66% (vs. 34% > 100 copies) ^§^ OS 82% (vs. 33% > 100 copies) ^§^	NA
			After consolidation	BM	*CBFB-MYH11/ABL* < 82 copies	CIR 32% (vs. 75% > 82 copies) ^§^ DFS 64% (vs. 25% > 82 copies) ^§^ OS 86% (vs. 25% > 82 copies) ^§^	
Yoon et al. [20], Bone Marrow Transplant. 2014	71 ^§^ 39 *	61.8 * NA	After induction	BM	MRD qPCR reduction > or = 3 log	Longer OS *	NA
			At the end of treatment	BM	MRD qPCR undetectable	Longer OS *	
Wang et al. [83], Ann. Hematol. 2014	10 ^§^ 40 ^§^	11.2 * CR 70% ^§^	4 weeks after 3rd consolidation	BM	*CBFB-MYH11/ABL* × 10^6^ < or = 0.1%	2-y RFS 56.8% (vs. 15.8% > 0.1%) *	NA
			4 weeks after last consolidation	BM	*CBFB-MYH11/ABL* × 10^6^ < or = 0.1%	2-y RFS 55.8%(vs. 25.4% > 0.1%) *	
			At follow-up	BM	*CBFB-MYH11/ABL* × 10^6^ < or = 0.1%	2-y RFS 75% (vs. 0% > 0.1%) *	
Qin et al. [84]., Leuk. Lymphoma 2015	86 34	25 CR 95.3% 3-y CIR 33.7% 3-y DFS 62.2% 3-y OS 72.9%	After course 1 induction	BM	*CBFB-MYH11* transcript levels > 2.0% (corresponding to <2-log reduction)	3-y CIR 68.5% (vs. 43.3% if *CBFB-MYH11* levels < or = 2.0%) 3-y DFS 31.5% (vs. 56.7% if *CBFB-MYH11* levels < or = 2.0%)	NA
			After achieving CR by induction	BM	*CBFB-MYH11* transcript levels > 2.0% (corresponding to <2-log reduction)	3-y CIR 77.6% (vs. 40.5% if *CBFB-MYH11* levels < 2.0%) 3-y DFS 22.4% (vs. 59.5% if *CBFB-MYH11* levels < 2.0%)	
			After course 1 consolidation	BM	*CBFB-MYH11* transcript levels > or = 0.2% (corresponding to < or = 3-log reduction)	3-y CIR 69.8% (vs. 7.1% if *CBFB-MYH11* levels < 2.0%) 3-y DFS 30.2% (vs. 92.9% if *CBFB-MYH11* levels < 2.0%) 3-y OS 48.9% (vs. 100% if *CBFB-MYH11* levels < 2.0%)	
			After course 2 consolidation	BM	*CBFB-MYH11* transcript levels > 0.2% (corresponding to < or = 3-log reduction)	3-y CIR 88.3% (vs. 26.9% if *CBFB-MYH11* levels < or = 0.2%) 3-y DFS 11.7% (vs. 73.1% if *CBFB-MYH11* levels < or = 0.2%) 3-y OS 26.9% (vs. 88.3% if *CBFB-MYH11* levels < or = 0.2%)	
Ishikawa et al. [28], Blood Adv. 2020	67 ^§^ 37 ^§^	52.2 * 2-y RFS 59.6% ^§^	At the end of consolidation	BM	*CBFB-MYH11* transcripts > or = 50 copies/μg RNA	Lower RFS ^§^	NA
Duan et al. [31], Br. J. Haematol. 2021	58 38	29.8 CR 98.3% 3-y CIR 29.4% 3-y CIM 24.4%	After 2^nd^ consolidation	BM	*CBFB–MYH11/ABL* < 0.1%	3-y RFS 100% (vs. 31.4% if *CBFB–MYH11/ABL* > 0.1%) 3-y EFS 100% (vs. 33.1% if *CBFB–MYH11/ABL* > 0.1%)	NA
Puckrin et al. [85], Haematologica 2021	47 ^§^ 46.5 *	44.4 * CR 99.1% *	At the end of consolidation	PB/BM	*CBFB-MYH11* transcripts reduction > or = 3 log	RFS 61.1% (vs. 33.7% if *CBFB-MYH11* transcripts reduction < 3 log) *	10^−4^
			At the end of treatment	PB/BM	*CBFB-MYH11* transcripts reduction > or = 4 log	RFS 51.2% (vs. 29.3% if *CBFB-MYH11* transcripts reduction < 4 log) *	
Duan et al. [32], Ann. Hematol. 2021	68 ^§^ 39 *	26 * CR 99.5% * 3-y CIR 29.4% * 3-y CIM 27% *	After 2nd consolidation	BM	*CBFB–MYH11*/*ABL* < 0.1%	3-y RFS 96.3% (vs. 34.6% if *CBFB–MYH11*/*ABL* > 0.1%) * 3-y OS 94.1% (vs. 51.3% if *CBFB–MYH11*/*ABL* > 0.1%) *	NA

CR: complete remission after induction; CR1: CR after first induction; OS: overall survival; RFS: relapse-free survival; EFS: event-free survival; DFS: disease-free survival; LFS: leukemia-free survival; CIR: cumulative incidence of relapse; CIM: cumulative incidence of mortality; CCR: continuous CR; PB: peripheral blood; BM: bone marrow; MRD: measurable residual disease; PCR: quantitative polymerase chain reaction; qPCR: quantitative PCR; B2M: beta2-microglobulin; GAPDH: glyceraldehyde-3-phosphate dehydrogenase; EoR: estimate of relapse; EoS: estimate of survival; NA: not available data; NS: not statistically significant data. * Data referred to entire cohort of the study. ^§^ Data referred to *CBFB-MYH11* AML cohort.

## Data Availability

Not applicable.
